# Progress and challenges of plant-derived nucleic acids as therapeutics in macrophage-mediated RNA therapy

**DOI:** 10.3389/fimmu.2023.1255668

**Published:** 2023-12-08

**Authors:** Yu-Da Liu, Hao-Ran Chen, Yao Zhang, Ge Yan, Hao-Jie Yan, Qi Zhu, Li-Hua Peng

**Affiliations:** ^1^ College of Pharmaceutical Sciences, Zhejiang University, Hangzhou, China; ^2^ State Key Laboratory of Quality Research in Chinese Medicine, Macau University of Science and Technology, Macau, China

**Keywords:** plant derived nucleic acids, cross-kingdom regulation, macrophage, pharmacological activities, delivery strategies

## Abstract

Plant-derived nucleic acids, especially small RNAs have been proved by increasing evidence in the pharmacological activities and disease treatment values in macrophage meditated anti-tumor performance, immune regulating functions and antiviral activities. But the uptake, application and delivery strategies of RNAs as biodrugs are different from the small molecules and recombinant protein drugs. This article summarizes the reported evidence for cross-kingdom regulation by plant derived functional mRNAs and miRNAs. Based on that, their involvement and potentials in macrophage-mediated anti-tumor/inflammatory therapies are mainly discussed, as well as the load prospect of plant RNAs in viruses and natural exosome vehicles, and their delivery to mammalian cells through macrophage were also summarized. This review is to provide evidence and views for the plant derived RNAs as next generation of drugs with application potential in nucleic acid-based bio-therapy.

## 1 Introduction

Plants contain a large number of active nucleic acids, which have been proved to have significant pharmacological activities and disease treatment values by increasing evidence. For examples, plant-derived miR2911 can resist influenza a virus (1), small RNA can reduce the viability of HeLa cells and promote their apoptosis (2), tRNA fragment derived from *Taxus Chinensis* was shown to inhibit ovarian cancer growth by targeting the transient receptor potential cation channel subfamily member 1 (3). However, researches on the immunologic mechanism of plant RNA therapy in mammals is limited.

**Figure 1:**
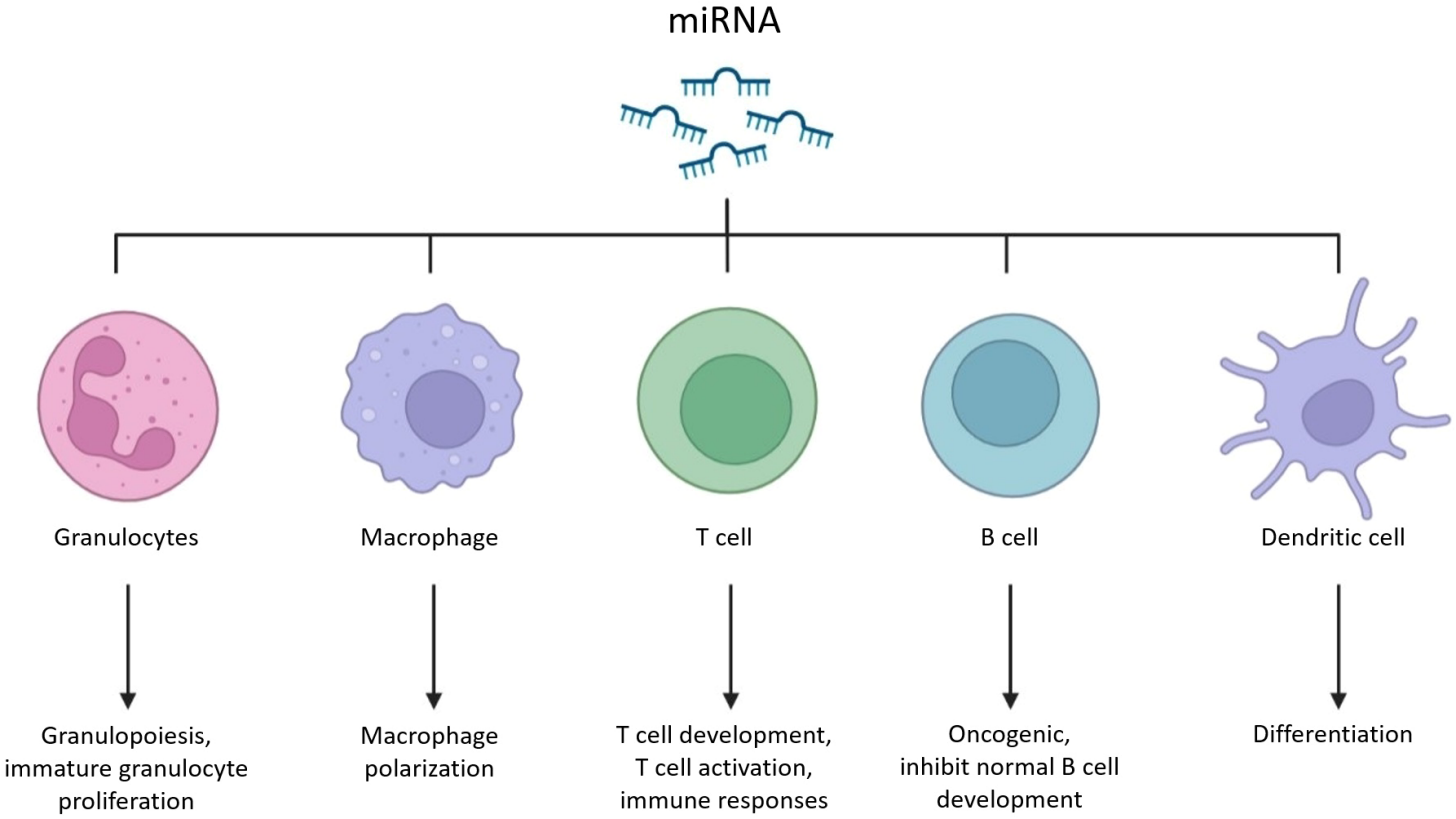
Schematic diagram of RNA meditated immune pathways.

Notably, in addition to involving in the development, internal stability and the regulation of many gene expression, mRNAs and miRNAs are more closely related to the macrophage-mediated immune responses, unlike the specific functions of siRNAs and tRNAs (**Figure 1**). It’s well known that activated macrophages can directly kill pathogens and tumor cells, but also participate in and regulate immune almost the whole responses, such as involving in antigen presentation to lymphocytes, T/B lymphocytes activation, and neutrophils recruitment. According to several studies, as one of the important regulators that can enter mammals, plant-derived RNAs has shown surprising therapeutic effects in macrophage-mediated immune system regulation. For instance, some RNAs such as soybean-derived gma-miR159a can reduce inflammatory infiltration of tumor environment by reducing over-activated macrophages induced inflammation (4), and another such as ginseng vesicles-derived RNAs can directly induce M1 macrophages to polarize and enhance the content of total ROS, thus accelerating the apoptosis of mouse melanoma cells (5). Besides, plant-derived RNAs can be directly involved in the immune process, such as the honeysuckle-derived miR2911 can inhibit tumor growth relies on functional T cells (6), and the miR156a from dietary green veggies reduces inflammatory cytokine-induced monocytes adhesion (7).

Therefore, summary of the activities and macrophage-mediated RNA therapies of plant-derived nucleic acids with potential as bio-drugs candidates and the related delivery technologies were necessary. This review therefore mainly summarizes the research progress about bioactivities and interactions of plants derived nucleic acids with mammalian cells, with their involvement in macrophage-mediated therapies are emphasized, as well as the delivery progress on them are reviewed. Based on that, the challenges and bottlenecks for the development of these natural nucleic acids into biodrugs for human diseases therapy are discussed and outlooked.

## 2 Cross-kingdom regulatory role of plant RNAs

Plant nucleic acids include about 30 types of ribonucleic acid (RNAs), including messenger RNA (mRNA), microRNA (miRNA), ribosomal RNA (rRNA), transfer RNA (tRNA), siRNA, etc. Some functional plant RNAs, such as mRNA, miRNA, and tRNA have been proven to play major cross-kingdom regulatory roles in mammalian gene expression to involve various physiological activities.

**Figure 2:**
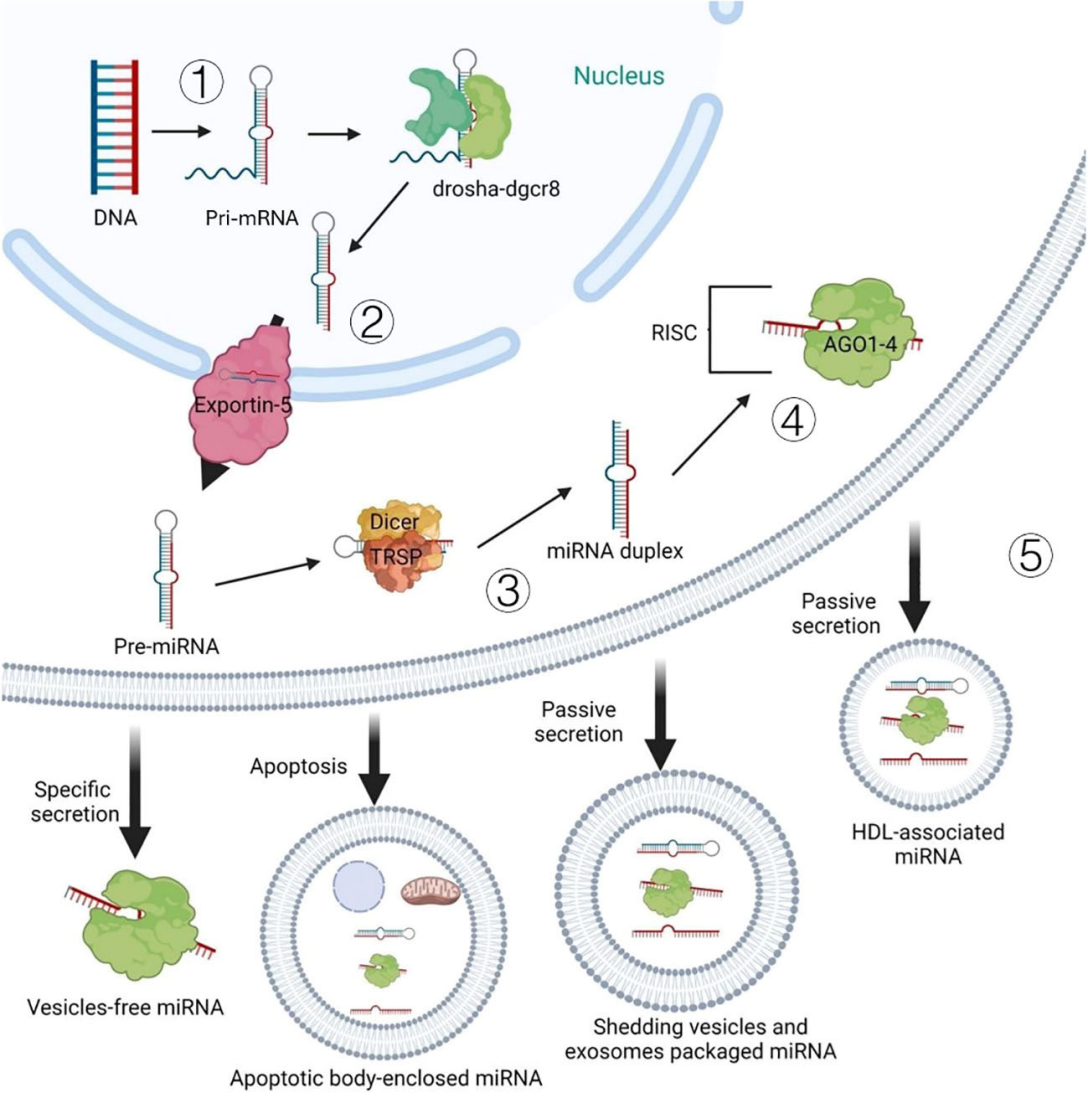
Intracellular biogenesis and extracellular pattern of miRNAs: ①the DNA encoding miRNA is transcribed into the pri-miRNA in the nucleus. ②pri-miRNA is further processed by the RNAse III enzyme drosha-dgcr8 complex to form pre-miRNA, which is transported from the nucleus to the cytoplasm through protein exportin-5. ③further processed into miRNA double-stranded by dicer enzyme and RNA binding protein TRBP. ④after the double-stranded miRNA is untied, the leading strand and argonaute protein form RNA-induced silencing comple.⑤Passive secretion ways for miRNA out of the cell (secreting exposed miRNA, apoptotic bodies, shedding vesicles and exosome-packaged miRNA).

mRNA is an active single-stranded ribonucleic acid carries genetic information that can guide protein synthesis (8), its localization contributes to the post transcriptional regulation of gene expression and controls the basic processes of cell migration, polarization and differentiation (9). And miRNAs as a kind of non-coding RNA can target and regulate post-transcriptional gene expression to involve various physiological activities by linking them with target mRNA (10, 11). The researchers were unexpected to find that there may also be some special mechanisms between animals and plants, which can enable plant mRNAs to play a cross-kingdom regulatory role. According to the discussion of Sulagna Das et al. at the level of different life kingdoms and sub organelles, the mechanism of mRNA localization and protein synthesis are highly conservative (12), and the active and passive transport of mRNA in animals and plants are very similar in this regard (13). Furthermore, previous studies have indicated that plant-derived miRNA can enter and stably existing in the blood and tissues of the mammal (14). More recent evidence suggests that extracellular miRNAs can also delivery in the form of vesicles such as apoptotic bodies, exfoliative vesicles and exosomes, and can bind to ago protein alone or high-density lipoprotein (**Figure 2**). These plant miRNAs are absorbed by cells of the mammalian digestive tract and packaged into micro vesicles, which protect them from degradation (15). Crucially, once plant-derived RNA enters the body of mammals, it can affect the physiological state of animals via regulating the host target genes (11). But we need more concrete evidence.

Specifically, Zhang L et al. indicated that rice-derived MIR168a is one of the most highly enriched exogenous plant miRNAs in the sera of Chinese subjects, this suggests that foodborne plant RNAs has indeed been stable in humans. And miR168a could bind to the human/mouse low-density lipoprotein receptor adapter protein 1 mRNA, inhibit its expression in liver, and consequently decrease LDL removal from mouse plasma (11). Besides, Gu et al. have proven that *Lycium barbarum L*-derived miR162a can pass through the gastrointestinal tract to target the bone marrow, and its transgenic *N. benthamiana* leaves effectively protected against osteoporosis in mice (16). The result suggest that plant-derived miRNAs can indeed play a cross-kingdom regulatory role through the gastrointestinal tract. It happens that there is a similar case, Li et al. found that plant miR167e-5p regulates the proliferation of enterocytes *in vitro*, significantly inhibited the proliferation of enterocytes in a dose- and time-dependent manner. And bioinformatics prediction and a luciferase reporter assay indicated that miR167e-5p targets β-catenin. In addition, the application of plant-derived tRNAs in mammal are rarely because its isolation from plants still remains a challenge. Recently, Yan et al. presented a new strategy to obtain tRNA of high integrity and purity from ginseng roots (17). Then Hu et al. reported the HC83 (a tRNA fragment from ginseng) protects heart against ischemia/reperfusion injury via targeting the lncRNA MIAT/VEGFA pathway, which provided the first evidence that plant-derived tRNA fragment can exert miRNA-like functions in mammalian systems (18). Accordingly, it can be predicted from these cases and related studies that miRNA plays key roles in the regulation of intraspecific and interspecific gene expression in almost all eukaryotes (including plants and mammals) (19), plant miRNAs may be a new class of officinal bioactive molecules for epigenetic regulation in humans and animals (20).

However, some scholars believe that the systemic delivery and regulation of plant-derived RNAs in humans is challenging, mainly because of a low miRNA level which was detected in mammalian tissues in some studies, and generally the control effects of miRNAs in plant and animal are different (21). In response to this problem, Mlotshwa et al. designed tumor suppressor miRNAs to mimic small RNAs produced in plants, and reported that oral administration of a cocktail of tumor suppressor miRNAs reduced tumor burden in the well-established ApcMin/+ mouse model of colon cancer. Also, higher concentration of these RNAs were detected in the miRNA-treated animals, these results suggest that the mimetic plant miRNAs were taken up by the digestive tract of ApcMin/+ mice upon ingestion (22). Therefore, the systemic delivery and regulation of plant-derived RNAs in humans are credible, its potential as therapeutics is well-founded, and have been confirmed by experiments and widely recognized.

However, up to now, only some representative mRNAs and miRNAs were found to play cross-kingdom regulatory roles in mammalian immunity and macrophage-mediated therapy, while the similar roles of plant-derived siRNAs, tRNAs, etc. are not in-depth studied.

## 3 Plant RNAs as therapeutics in macrophage-mediated therapy

At present, the therapeutic nucleic acids derived from plants are rich in resource, the effects of plant RNAs in treating human diseases have been reported, such as tumors, the chronic idiopathic urticaria (23), double allele RPE65 associated retina, malnutrition, spinal muscular atrophy, multiple peripheral nerve diseases, acute liver frightening disease, severe combination, etc. (**Table 1**) (33). Among them, it is noticed that plant-derived nucleic acids have unique therapeutic applications in macrophage-mediated anti-tumor effects, immune regulating functions, antiviral activities and regulation of cells apoptosis.

**Table 1:** List of the reported plant nucleic acids with therapeutic effects.

Plants	Names	Functions	Mechanisms
**Honeysuckle** (1)	miR2911	Sars-cov-2 replication inhibition	Inhibits protein translation by binding to SARS-COV-2 genome.
**Honeysuckle** (6)	miR2911	Repress colon tumor development	Strongly binding TGF-β1 mRNA, down-regulating TGF-β1 expression.
**Arabidopsis** **thalia** (24)	miR159	Breast tumor growth inhibition	Reduce MYC protein levels by targeting the TCF7 transcription factor encoding the Wnt pathway.
**Soybean** (25)	gma-miR159a-3p	Colon cancer inhibition	Suppressed the expression of the oncogene MYC downstream of the Wnt signaling pathway by targeting the TCF7 gene.
**Ubiquitous** (26)	miR171vr	Treating oral cancer, breast and prostate adenocarcinoma	Significantly reduced human G protein subunit α 12 mRNA and protein levels.
**Olea europaea** (19)	oeu-sR20, oeu-sR27, oeu-sR34	Affects tumor cell viability and apoptosis	Similar to hsa-miR34a, significantly decrease SIRT1 and BCL2 proteins expression.
**Licorice** (27)	**/**	Improves immune function	Induce significant proliferation and aggregation of human peripheral blood mononuclear cells to improves immune function.
**Ginger** (28)	mdo-miR7267-3p	Ameliorate mouse colitis	mdo-miR7267-3p-mediated targeting of the LGG monooxygenase ycnE yields increased indole-3-carboxaldehyde. Induce production of IL-22 to barrier function improvement.
**Longan** (29)	clo-miR-14	inflammatory	Highly similar to human miRNA (hsa-miR4693-5p) and has high serum stability.
**Rhodiola crenulate;** **Taraxacum mongolicum** (30)	HJT-sRNA-m7; PGY-sRNA-6	Anti-fibrosis and anti-inflammatory effects, respectively	**/**
**Strawberry** (31)	miR168	Ameliorate autoimmune Encephalomyelitis; Inhibition of T cell proliferation	Interacts with dendritic cells by binding to the outer domain of TLR3; Limiting dendritic cell migration and dampening Th1 and Th17 responses.
**Garlic** (32)	Exosome RNAs	Target the brain inflammation of obesity mice	Through IDO1 mediated AHR pathway and c-Myc mediated c-GAS/STING inflammatory pathway.

/, Lack of relevant information in the study.

### 3.1 Plant RNAs in anti-tumor and immune regulation

Studies have shown that some plant nucleic acid molecules can interact to animal to exhibit anti-tumor functions. This is because a high degree of similarity has been found on the molecular basis of RNA biogenesis and action between plants and animals. Some plant-derived miRNAs are ingested by animals and can exert their gene regulatory effects across domains via macrophages or other pathways. These plant miRNA targeted genes are involved in cancer inhibition (34).

The transport and presentation of plant RNAs to cells is critical. Chin et al. find that western donor serum contains plant miR159, these plant miR159 are detected mainly in extracellular vesicles (24). However, their further sequencing data as well as others have observed that plant miRNAs that are taken up after consumption are not necessarily those that are most abundant in the plant (11, 35). Therefore, we were curious that there is likely a selective mechanism for the uptake of specific plant miRNAs in mammals.

In the certain research of Liu C et al, various results showed miR2911 relies on functional T cells to exert its antitumor activity. Mechanistically, miR2911 reversed the tumor-promoting effect of TGF-β1 by an increase of T lymphocytes infiltration, in other words, miR2911 relied on functioning T cells to exert its anti-tumor effect (6). RNA is an intrinsically safe vector because it is the smallest and only transient information carrier that does not interact with the genome (36). But such information carriers also need to be firstly ingested and presented. We know that macrophages act as antigen-presenting cells to present bioinformation to T lymphocytes to promote immune responses against specific pathogens, so it is possible to assume that miR2911 is also likely to achieve anti-tumor and immune function through the uptake and presentation of macrophages.

Minutolo et al. found that Olea europaea Linn miRNAs (19), oeu-sR20, oeu-sR27 and oeu-sR34 have great potential to be used as novel, natural non-toxic, anti-cancer therapeutics. By the way, a series of transfection experiments were performed on lymphoid and monocytoid cells, and the effective ability of synthetic sequences of oeu-sRs to modulate the protein expression of hsa-miR34a-specific targets (SIRT1, BCL2 and SNAIL) were verified, it also indicates the potential in regulating the corresponding targets in monocyte-associated macrophages.

On the other hand, selectively presented plant-derived RNAs kill tumor cells also through the macrophages. RNAs can reduce inflammatory infiltration of tumor environment by reducing over-activated macrophages induced inflammation. CD11b is involved in various adhesion interactions of monocytes, macrophages and granulocytes. Notably, dysregulation of miRNAs has been found in many tumors including breast cancer (37, 38). According to Liu J et al, gma-miR159a, which is abundant in soybean, not only inhibit the breast tumors, but also significantly reduced the number of CD11b-positive cells, the expression of CD11b gene, and the infiltration of immune cells (4). Extracellular vesicles (EVs) isolated from ginseng roots can induce M1 like macrophages to polarize through Toll like receptor 4/myeloid differentiation antigen 88 signal pathway, and enhance the content of total ROS, thus accelerating the apoptosis of mouse melanoma cells (5). These studies provide novel evidence for the macrophage-mediated anti-tumor effects of plant nucleic acids.

The existence of heterogeneous miRNAs has a cross-border anti-tumor effect, which might be based on their key influence in inflammatory environment (24). In the development of nucleic acid vaccines, it was accidentally discovered that injection of mRNA resulted in localized protein expression and immune responses against the encoded antigen (39), mRNA allow for the simultaneous delivery of multiple messages, including various TAA (Tumor Associated Antigen) or somatic tumor mutations, eliciting humoral and cell-mediated immune responses (40). On this basis, it has been noticed that miRNAs are more closely related to immune responses, such as innate immune responses, T and B lymphocyte differentiation, pathogen infection, and immune and inflammatory immune regulation, unlike the gene silencing effects of siRNAs (41). Such as the ectopic expression of miR156a from dietary green veggies in human aortic endothelial cells reduces inflammatory cytokine-induced monocytes adhesion by suppressing junction adhesion molecule-A (7). As an important regulator of mammalian immune system, miRNA is involved in development, internal environmental stability and regulation of many pathways. These effects must not be bypassed by immune cells such as macrophages, and even plant-derived miRNA can play an unexpected role. In a study of mouse breast cancer, plant miR159 was found to inhibit the growth and proliferation rate of MDA-MB-231 by targeting TCF7, which encodes a Wnt signaling transcription factor. The interaction between Wnt pathway and macrophages also played a considerable role.

For example, Inujima et al. found a heat-resistant RNA with a 90-amino acid sequence in a decoction of *Glycyrrhizae Radix*, which activated NF-κB/AP-1 and induced TNF-α production in murine macrophages (42). Research shows that apple derived EVs also have anti-inflammatory properties, with the mechanism is relevant to that miR-146a-5p in EVs can regulate the NF-κB (nuclear factor-κB) pathway, decreasing the expression of human proinflammatory cytokines (such as IL-8 and IL-1 secreted by macrophages) (43).

Also, for the treatment of lung inflammation in coronavirus disease 2019, Teng et al. show that miRNA aly-miR396a-5p from ginger exosome-like nanoparticle abolished the induction of exosomes^Nsp12Nsp13^-mediated lung inflammation which released from the lung epithelial cells in lung macrophages. Mechanistically, the activation of macrophage-associated nuclear factor κB (NF-κB) and the release of an array of inflammatory cytokines such as TNF-α, IL-6, and IL-1β are inhibited (44).

Furthermore, mRNA has been found to be taken up directly by immune cells such as macrophages, dendritic cells, and neutrophils to activate T cells, in addition to entering lysosomes (mostly) and cytoplasm (a little) via endocytic pathways (39). Studies have shown that miR-21 is transmitted to macrophages, silencing PTEN and PDCD4, promoting the production of IL-10 and thus playing an anti-inflammatory role. Similarly, plant miRNAs with similar targets can also play anti-inflammatory effects after phagocytosis by macrophages (45).

### 3.2 Plant RNAs in antiviral therapy

The polarization of macrophages towards M1 phenotype is important for effective antiviral immune response. At the same time, the reduction of inflammatory damage in histiocytes requires M2 polarization. At present, many studies have shown that plant-derived EVs, polysaccharides, enzymes and other particles and active molecules regulate the polarization of macrophages and play an antiviral role. However, the direct regulation of the activation and polarization process of macrophages by plant-derived RNAs are not clear, which may be due to the fact that a single type of RNA cannot effectively achieve and regulate the balance of polarization. Moreover, the interactions of multiple RNA species are difficult to parse. Therefore, the current research on the antiviral effect of plant RNAs is mainly focused on its direct targeting and silencing effect to viruses.

**Figure 3:**
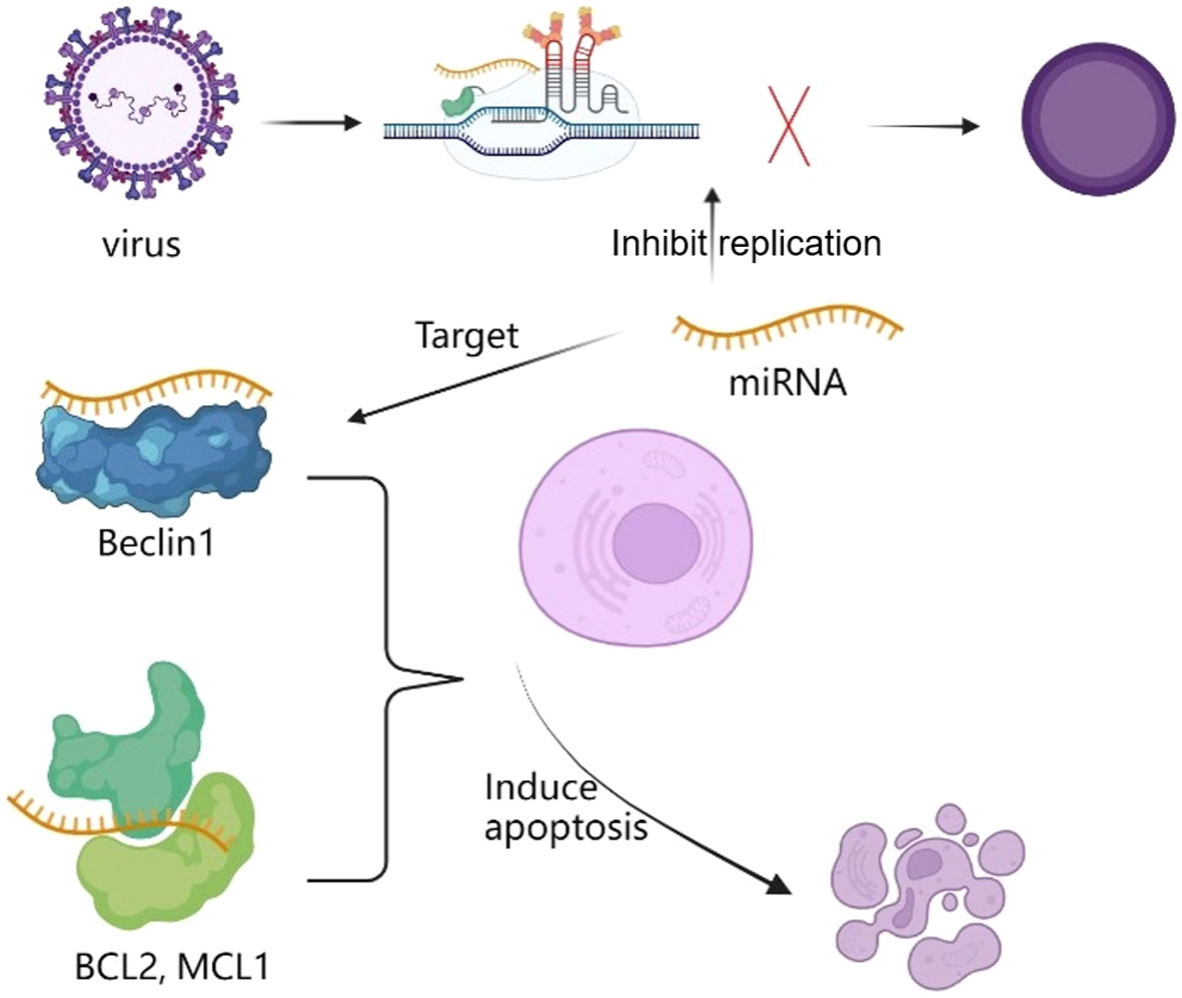
miRNA regulate the anti-apoptotic proteins (BCL2, MCL1) and autophagy promoting protein Beclin1 interactions to inhibit replication and induce apoptosis to virus.

In general, RNA has the potential for anti-antiviral role by inhibiting viral replication, key protein expression and inducing apoptosis. Take apoptosis as an example, studies have found that many RNAs such as miRNAs can regulate the anti-apoptotic proteins (BCL2, MCL1) and autophagy promoting protein Beclin1 interactions to regulate autophagy and apoptosis, so as to interfering with replication and inducing virus apoptosis (**Figure 3**). Currently, some plant-derived miRNAs have been found to target viral genome and proteins to inhibit viral replication (46). For example, two plant-derived small silencing RNAs (amiR471 and amiR519) were found in edible lettuce, and these miRNAs were found to inhibit HBsAg expression.

There are already some representative medicinal plants in antiviral therapy. The atypical miR2911 encoded by honeysuckle is considered to be the first active plant miRNA found in traditional Chinese medicine and gets much attention, which can inhibit the replication of influenza A virus, including H1N1, H5N1 and H7N9. The antiviral activity of miR2911 can be attributed to its ability to bind to the nucleotide sequences of Pb2 and NS1 genes encoded by H1N1 virus (35). These results taken the lead to identify miR2911 as the active component that directly targeting influenza A viruses, which has firstly confirmed the natural product could directly target the virus. But also, it was confirmed that the physiological concentration of miR2911 in Honeysuckle decoction is enough to resist IAV, and it is further confirmed that following the entrance into the body of animal exogenous miRNA has been respectively transported to tissues through diet, and the concentration is sufficient to generate physiological function. In addition, it was found that hd-miR2911 shortened the time for male and female patients to become SARS-COV-2 PCR negative (1).

It was found that miR2911 derived from Honeysuckle directly inhibits Enterovirus 71 replication via targeting VP1 gene (47). On the one hand, the results of gene analysis and western blot analysis showed that miR2911 significantly inhibited the expression of VP1 protein of EV71 virus. On the other hand, RNA-binding protein immunoprecipitation experiments demonstrated the association of VP1 mRNA with Ago2. Besides, they selected several clinically isolated EV71 viruses for further evaluation, with the results demonstrating that miR2911 has effective antiviral activity against various EV71 strains. Similarly, Ying Huang et al. reported that Honeysuckle-derived miR2911 directly inhibits varicella-zoster virus replication by targeting IE62 gene (48).

In addition, advanced scientific tools have been used to screen plant nucleic acids for antiviral activity. Aiming to contain outbreaks such as the COVID-19, Mangukia N et al. designed a systematic computational workflow to identify the cellular miRNAs from human host possessing the capability to target and silence 3’UTR of SARS-CoV-2 genome. And base on this, they predicted that the members of miR477 family commonly found in *Ocimum Tenuiflorum*, Zingiber officinale and Piper nigrum genomes possess an inherent potential to silence viral genome RNA and facilitate antiviral defense against SARS-COV-2 infection (49). All these results remind the great potential of plant miRNAs as drug candidates for the inhibition of virus replication.

## 4 Delivery strategies for plant nucleic acids

**Figure 4:**
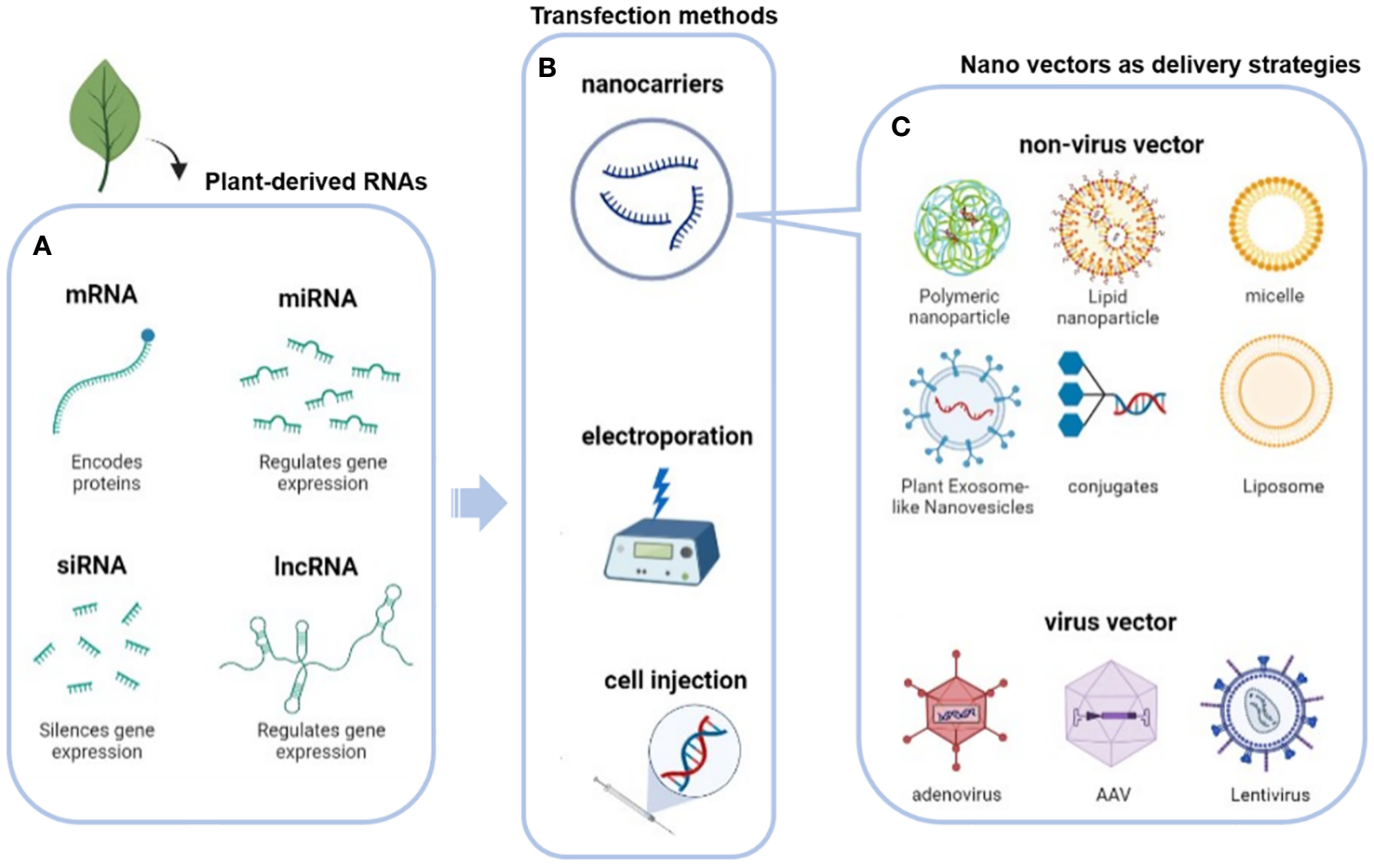
Delivery strategies for plant nucleic acids: **(A)** Plant-derived RNAs and their main functions; **(B)** transfection method (nano-vectors, physical transfection such as electroporation and cell injection); **(C)** virus or non-virus nano vectors as plant-derived RNAs delivery strategies.

As biological macromolecule, many physical and chemical properties such as unstable under external environment and the large polarity, nucleic acid are not advantageous for delivery, resulting in its low bioavailability and limited application potential in clinic. These limitations require the use of appropriate carriers to improve bioavailability and optimize therapeutic efficacy (50). To address these issues, potential viral vectors and novel plant-derived EVs have been proposed (**Figure 4** and **Table 2**).

**Table 2:** Delivery systems and features.

Delivery systems	Cargo Loading Techniques	Features
**Solid Lipid nanoparticles** (51)	Electrostatic complexation	High transfection efficiency and better biocompatibility.
**Polymers** **Nanoparticles** (52)	Through hydrophobic interaction, electrostatic interaction	High surface volume ratio, low toxicity, strong absorption capacity
**Micelle** (53)	Self-assembly to form core-shell structure	Reduced MPS clearance and higher brain targeting ability
**Liposomes** (54)	Lipid bilayer structure similar to cell membrane	High stability, liver and spleen targeting, sustained release and detoxification
**Nanosuspension** (55)	Submicron colloidal dispersion system formed by surfactants	High drug loading, small side effects and convenient amplification
**Plant Exosome-like nanovesicles** (56)	Cross-kingdom delivery and regulation	High bioavailability, non-immunogenic and innocuous traits
**Conjugates** (57)	Chemical coupling	High stability and delivery targeting.
**Ad as a vector** (58)	Attenuated or non-toxic virus plasmids.	Transient expression, large cargo capacity, high transduction but high immunogenicity
**AAV as a vector** (58)		Lower innate and viral immunity, small cargo capacity
**Lentivirus vector** (58)		Allowing long-term transgene expression, high packaging capacity, weak immunogenicity

### 4.1 Viral vectors as delivery strategies

Numerous viral vectors delivery systems can be constructed using macrophage-based immune cells as specific targets. Specifically, the macrophages and hepatocytes are considered as potential target sites in the area of drug discovery and drug delivery as they together play an important role in various infectious diseases (59). Antigen-presenting cells such as macrophages and dendritic cells can absorb virus particles through scavenger receptors and trigger a strong cellular immune response.

Miyazaki Y et al. believe that the potent activity of specific miRNAs as disease modifiers both *in vitro* and *in vivo*, and they described a novel therapeutic approach using the AAV vector–mediated delivery of a specific miRNA for spinal and bulbar muscular atrophy (SBMA), the early intervention of miR-196a delivered by an AAV vector ameliorated the phenotypes of SBMA in a mouse model. And miR-196a enhanced the decay of the androgen receptor mRNA by silencing CUGBP, Elav-like family member 2 (CELF2). CELF2 directly acted on androgen receptor mRNA and enhanced the stability of androgen receptor mRNA (60). Based on this evidence, specific known sequences of RNA can be targeted by viral vectors, so it is bold to assume that identified plant-derived active nucleic acids can also be designed to achieve interspecific delivery and specific expression by viral vectors, but unfortunately, few related studies have been reported.

### 4.2 Plant-derived EVs as delivery nanoplatforms

Although viral vectors are good at delivering genes, they are likely to cause a variety of unexpected immune responses, so their practical application has been greatly limited. Another route, non-viral vectors has been developed, one is synthetic nanoparticles, including micelle, conjugates and liposome; another one is natural plant-derived EVs, which mainly delivery nucleic acids through their unique physicochemical properties and engineered targeting capabilities. Plant EVs also contain multiple types of natural RNAs, are ideal natural RNA delivery vehicles *in vitro* and *in vivo*. These vectors enter cells by endocytosis or non-endocytosis, deliver nucleic acid drugs to target sites, and then exert therapeutic effects (61). In studying plant-derived EV’s passage through the harsh gastrointestinal environment and into intestinal cells intact, it was found that grapefruit derived EVs loaded with methotrexate was able to target F4/80+ macrophages in the intestinal lamina propria via micropinocytosis or cellular uptake pathways. Based on this, miRNAs in grapefruits derived EVs were found target macrophages and enhance their anti-inflammatory capabilities, thereby maintaining intestinal immune homeostasis. In addition, the unique targeting of plant-derived EVs is constantly being discovered, such as grape derived EVs targeting gastrointestinal cells, ginger (62) and mulberry bark derived EVs also targeting macrophages to reduced acute colitis, enhanced intestinal repair, and prevented chronic colitis and colitis-associated cancer. This shows the great promise of plant-derived EVs (56). More importantly, we are concerned that there are very few reports on the delivery of plant nucleic acids by synthetic nanoparticles, a key point is that EVs as endogenous substances from plants which mediate signal transduction, can achieve better delivery and therapeutic effects as natural carriers of plant autologous nucleic acids.

It has been reported that ginseng-derived exosomes (GExos) can deliver the loaded miRNAs into bone marrow mesenchymal stem cells (BMSCs), thereby stimulating the growth of BMSCs and neural differentiation (63). Beyond that, another study about GExos demonstrated the uptake of GExos in macrophages and its role in promoting polarization (5). The M1/M2 ratio of tumor-associated macrophages correlating with tumor growth, angiogenesis and invasion, Cao M et al. analyzed associated surface markers, genes and cytokines of macrophages treated with GExos, the GExos indeed significantly promoted the polarization of M2 to M1 phenotype. Although they claimed that the introduction is largely dependent on TLR4 and MyD88 signaling from ceramide lipids and proteins of GExos, the successful uptake of GExos in macrophages still demonstrates the feasibility of RNAs delivery. In conclusion, considering that plant nucleic acid has become an effective therapeutic cargo, the role of these natural exosomes carrying plant nucleic acid as a delivery vehicle is also recognized.

## 5 Discussion

In recent years, more and more research on medicinal plant nucleic acid demonstrated the advantages of plant nucleic acids in large genes bank, easy availability and low toxicity, forming a potential treasure house for gene therapy. Today, increasing studies have shown the successful regulation of plant small RNAs in mammals is not accidental. Since the discovery of honeysuckle-derived miR-2911 in directly targeting influenza A virus (35), more functions have been discovered one after another in plant derived nucleic acids, such as the targeting to VP1 gene to inhibit the replication of enterovirus type 71 (47), the direct inhibition of varicella-zoster virus replication (48), and suppression in the transforming growth factor-β1 medicated tumor growth (64). However, the RNA content and species are quite complex in different plants, research on plant RNAs as bio messages is still less mature and in-depth than that on animal nucleic acids, the standardized and systematic extraction technology, the mechanism of generating pharmacological activity and the novel and efficient delivery strategy are all bottleneck problems that need to be solved. The establishment of systematic plant nucleic acid gene library is highly suggested. Further, RNA localization technology to improve the targeting of plant nucleic acids in human body, as well as the nucleic acid protein hybridization are proposed to be used to improve the stability of nucleic acid drugs *in vivo* and prolong their action cycle.

Furthermore, most current studies have focused on the direct efficacy of plant RNAs on disease target cells, but in terms of uptake of plant RNAs in animal cells and immune-mediated signaling, the presentation of macrophages mentioned in existing studies may be a key step in effective delivery that is easily overlooked. Even in mammalian MSC-derived extracellular vesicles (MSC-EVs), the MSC-EVs-based anti-inflammatory effects were relied on the delivery of immunoregulatory miRNAs and immunomodulatory proteins in inflammatory immune cells (M1 macrophages, dendritic cells, CD4+Th1 and Th17 cells), enabling their phenotypic conversion into immunosuppressive M2 macrophages, tolerogenic dendritic cells and T regulatory cells (65).

Given the apparent roles of immune cells in physiological activities, much more exploration is deserved to investigate the potential of plant nucleic acids as immunological regulators in stimulating macrophages meditated immune therapy in such as cancer, inflammation, virus therapy and refractory diseases. Understanding in the working mechanisms of these exogenous plant RNAs is critical to developing they into new bio-drugs. This review provides a platform of the current state of knowledge of plant RNAs as therapeutics, especially in macrophage-mediated interference in diseases, which may also provide a unique approach to expanding the knowledge of the related interdiscipline fields.

## Author contributions

Y-DL: Data curation, Formal analysis, Investigation, Methodology, Supervision, Validation, Writing – original draft, Writing – review & editing. YZ: Data curation, Formal analysis, Investigation, Methodology, Writing – original draft. GY: Formal analysis, Investigation, Writing – original draft. H-RC: Data curation, Investigation, Methodology, Writing – original draft. H-JY: Formal analysis, Writing – original draft. QZ: Formal analysis, Writing – original draft. L-HP: Conceptualization, Funding acquisition, Resources, Supervision, Validation, Writing – review & editing.
